# Properties of FeAlSi-X-Y Alloys (X,Y=Ni, Mo) Prepared by Mechanical Alloying and Spark Plasma Sintering

**DOI:** 10.3390/ma13020292

**Published:** 2020-01-08

**Authors:** Filip Průša, Olga Proshchenko, Andrea Školáková, Vojtěch Kučera, František Laufek

**Affiliations:** 1 Department of Metals and Corrosion Engineering, University of Chemistry and Technology, Technická 5, 166 28 Prague, Czech Republic; proscho@vscht.cz (O.P.); skolakoa@vscht.cz (A.Š.); kucerao@vscht.cz (V.K.); 2 Institute of Physics, The Czech Academy of Sciences, Na Slovance 1999/2, 182 00 Prague, Czech Republic; frantisek.laufek@geology.cz; 3 Czech Geological Survey, Geologická 6, 152 00 Prague, Czech Republic

**Keywords:** mechanical alloying, spark plasma sintering, hardness, compressive strength, oxidation resistance, wear

## Abstract

Short-term mechanical alloying and compaction by spark plasma sintering was used for the production of FeAl20Si20Mo20-XNiX (X corresponds to 5–15 wt %) alloy, which showed an ultrafine-grained microstructure with dimensions of phases around 200 nm or smaller. It was found that the addition of Mo and Ni to the FeAl20Si20 alloy results in the formation of the AlMoSi phase compared to the three-phase FeAl20Si20 alloy, which initially contained FeSi, Fe_3_Si, and Fe_3_Al_2_Si_3_ phases. All the investigated alloys increased their hardness, reaching up to 1401 HV 1 for the FeAl20Si20Mo5Ni15 alloy, which contained in total 58.5% of the FeSi and Fe_3_Al_2_Si_3_ phases. As a result, all the prepared alloys showed one order magnitude lower wear rates ranging from 3.14 to 5.97·10^−6^ mm^3^·N^−1^·m^−1^ as well as significantly lower friction coefficients compared to two reference tool steels. The alloys achieved high compressive strengths (up to 2200 MPa); however, they also exhibited high brittleness even after long-term annealing, which reduced the strengths of all the alloys below approximately 1600 MPa. Furthermore, the alloys were showing ductile behavior when compressively tested at elevated temperature of 800 °C. The oxidation resistance of the alloys was superior due to the formation of a compact Al_2_O_3_ protective layer that did not delaminate.

## 1. Introduction

The Fe–Al–Si-based alloys belong to a perspective group of materials that was developed by joining two binary alloy systems, namely Fe–Al and Fe–Si. These alloys might find their utilization as a much cheaper and lighter substitution of heat-resistant steels or even nickel superalloys [1,2]. The main advantages of these alloys are their excellent thermal stability and resistance against high-temperature reactions in oxygen or sulfate-bearing atmospheres [3,4,5,6,7]. Such behavior has been initially described for the Fe–Al system, which is capable of maintaining its mechanical properties up to 500 °C [8,9]. Simultaneously, the Fe–Al alloys create a compact and protective layer made of either of α-Al_2_O_3_ or γ-Al_2_O_3_, whose formation is temperature-dependent [2,10]. Comparing these two modifications, the latter mentioned provides significantly better protection, since it does not contain pores as the α-Al_2_O_3_ does [1]. 

The present oxidic layer acts as a shielding bipolar membrane that decreases the diffusion of metal atoms through the layer toward the environment while also blocking the gases’ transport in the opposite direction. The α-Al_2_O_3_ modification provides lower protection since it contains many pores and is often prone to the formation of microcracks. Notably, the temperatures between 800 and 900 °C are known to be responsible for these kinds of defects. In this temperature interval, a metastable θ-Al_2_O_3_ is often formed and further transforms during cooling into a stable α-Al_2_O_3_ form, which is accompanied by a volume change [11]. As a result, tensile stresses are induced into the layer, allowing the formation of microcracks while simultaneously decreasing the adhesion of the layer to the alloy surface [12]. 

The negative effect of high porosity can be overcome by the addition of Si due to a formation of far more complex and dense compounds called spinels. Especially, the low addition of Si can effectively reduce the porosity of the protection layer. However, when some critical content of Si is exceeded, the porosity increases again, e.g., the Fe–Al–Si alloys containing over 30 wt % Si are exhibiting enormous porosity, making them unusable [10,13,14,15,16,17]. Simultaneously, the addition of Si toward the Fe–Al alloys suppresses the formation of aluminides in favor of silicides or alumino–silicides. Thus, e.g., the FeAl20Si20 (wt %) alloy is composed of FeSi, Fe_3_Si and Fe_3_Al_2_Si_3_ [15], or FeAl_2_Si phases [13,18]. The substitution of aluminides by silicides increases the hardness and strength of alloy as well as the wear resistance and thermal stability, although at the expense of toughness [9,10,13,15,16,19,20,21,22]. It has been found that the content of various forms of silicides determines the hardness of these alloys. The major contribution to the resulting hardness is mainly caused by the FeSi phase, which is known for its high hardness of 958 HV 1 [19]. Accordingly, to the work of Wu et al. [23], the FeSi phase exhibits the third-highest calculated hardness among the phases present in the Fe–Si binary system. According to this, the FeAlSi alloys were reported to exhibit the hardness of 730 HV 5 in case of FeAl10Si30 alloy [13,14,15]. On the other hand, the increase in Al content at the expense of Si results in a hardness decrease down to 440 HV 5 in the FeAl30Si10 alloy. 

A further increase in the hardness and strength of the FeAlSi alloys may be achieved by the alloying of transition elements, especially Ni and Mo. Up to now, almost no reports have mentioned the influence of these elements on the microstructure and properties of quaternary and foremostly quinary alloys. Novák et al. [24] reported that the addition of Ni in the FeAl20Si20 (wt %) alloy prepared by reactive synthesis resulted in a decrease of porosity while increasing the hardness and wear resistance as well as thermal stability and oxidation resistance at 800 °C. The same alloy, although prepared by mechanical alloying (MA) and compacted via spark plasma sintering (SPS), showed quite similar results, increasing the hardness and compressive strength from 1049 HV 1 up to 1376 HV 1 and from 1085 MPa up to over 1800 MPa [25].

The Fe–Al–Si-based alloys are in general prepared by powder metallurgy, which overcomes the problems experienced during cast-metallurgy processes. Especially, the MA is capable of providing and retaining the beneficial microstructural refinement and homogeneity, which further improves the mechanical properties [26,27,28,29,30]. During the process, a highly localized cold welding allowing only a limited diffusion of elements; continuous fracturing and deformation strengthening is responsible for the ultrafine or even nanocrystalline microstructure of the alloys [27,31,32]. As a compaction method, various techniques including uniaxial pressing and isostatic pressing, both done at elevated temperatures, seem to be failing to deliver expected results. The main setback is a long duration of the processes allowing microstructural coarsening, which deteriorates the desirable properties gained by the MA [33]. Thus, the fast compaction via SPS is capable of providing almost full-density compacts and is especially of interest. 

Thus, the present work describes the influence of Ni and Mo addition onto the complex properties of FeAl20Si20 alloy prepared by a combination of mechanical alloying and spark plasma sintering. The aim was to describe the effects of the different amounts of Ni and Mo as alloying elements on the microstructure, mechanical properties, and thermal stability, including oxidation resistance. 

## 2. Materials and Methods 

The FeAl20Si20Mo20-XNiX (X = 5–15 wt %) alloys were prepared from pure elements, which were mixed in appropriate amounts forming 20 g powder batches for mechanical alloying (MA). For this purpose, powders of Fe (purity of 99.9%, Strem Chemicals, Newburyport, US), Al (purity of 99.7%, Strem Chemicals, Newburyport, MA, US), Si (purity of 99.5%, Alfa Aesar, Lancashire, UK), Mo (purity of 99.5%, Alfa Aesar, Lancashire, UK), and Ni (purity of 99.5%, Merck, Darmstadt, United Kingdom) were used. The powders were placed into a milling jar together with milling balls, which were both made from AISI 420 stainless steel. Afterwards, the jar was sealed and flushed with Ar (purity of 99.996%) for 2 min with a constant flow of 2 l/min. Mechanical alloying was done in a milling device Retsch PM100 CM (Retsch, Haan, Germany) for 10.5 h while for each 30 min of the process, a short 10-min pause was maintained to suppress the excessive cold welding. 

Then, prepared powders were compacted via spark plasma sintering (SPS, FCT Systeme, HP-D 10, Rauenstein, Germany) using a heating rate of 300 °C/min until reaching 900 °C, after which the heating rate was reduced to only 100 °C/min. The samples were compacted at a temperature of 1000 °C with a pressure of 48 MPa and remained at this temperature for 10 min. Afterwards, the samples were slowly cooled down to 300 °C with a speed of 50 °C/min to reduce the thermal stress–strains within the sample. Prepared samples were cut using a diamond blade cutting device (Leco Precision VC-50 Vari-Cut, St. Joseph, US) into samples which were either used for microstructural investigations of for mechanical testing. 

Present phases were determined by powder X-ray diffraction (XRD, Bruker D8 Advance, Karlsruhe, Germany, Cu*K*α radiation and LynxEye-XE detector), while the actual chemical composition of the prepared samples was determined by X-ray fluorescence analysis (XRF, ARL 9400 XP, Thermo ARL, Switzerland). Semi-quantitative phase analysis, as well as the calculation of lattice and microstructural parameters were performed by the Rietveld method using the Topas 5 program (Bruker AXS, 2014). The microstructure of the prepared cross-sections was investigated using scanning electron microscopy (SEM, Tescan Lyra, Brno, Czech Republic) equipped with an energy-dispersive spectrometer (EDS, Oxford Instruments, 80 mm^2^, High Wycombe, United Kingdom). The surface porosity was determined using light microscopy (LM, Olympus PME-3, Tokyo, Japan), obtaining at least 20 micrographs with a total area of 0.55 mm^2^, which were then analyzed by the threshold method.

For the compressive tests, cuboid samples with a height length corresponding to 1.5 times the length of the bottom side were used. The compressive tests were done on a universal testing device (LabTest SP 250.1-VM, Labortech s.r.o., Opava, Czech Republic) with a strain speed of 0.001 s^−1^. Prepared alloys were also tested for thermal stability, which was determined by the hardness change during long-term annealing and by compressive tests, which were done either at laboratory temperature after annealing or at an elevated temperature of 800 °C. 

Furthermore, the samples were also investigated for the kinetics of cyclic oxidation at 800 °C. For this purpose, the samples were placed into the electric resistance furnace for time segments composed of 4, 9, 25, 50, 75, and 100 h, and then cooled down outside the furnace. The weight gain due to a formation of oxidic products was measured on an analytical balance (Pioneer PA224, Ohaus, Parsippany, NJ, US). 

To fully describe the mechanical properties, tribological tests were done using a pin-on-disc setup (TRIBOtechnic, Clichy, France). The tests were done on polished samples at laboratory temperature in an oscillating regime with an Al_2_O_3_ ball 6 mm in diameter that was moving with a speed of 10 mm·s^−1^ until reaching a total distance of 15 m. The ball was loaded with 5 N, and the wear track profile has been measured with a profilometer. The temperature and humidity during the tests were constant during all the tests corresponding to 22.2 °C and 35.5%. Obtained results were compared with the results of tool steels 1.2379 (AISI D2) and 1.3343 (AISI M 2) supplied from an external company. Both the steels were heat-treated by the supplier accordingly to the conditions specified in the relevant standards.

## 3. Results and Discussion

### 3.1. Phase Composition and Microstructure

**Figure 1:**
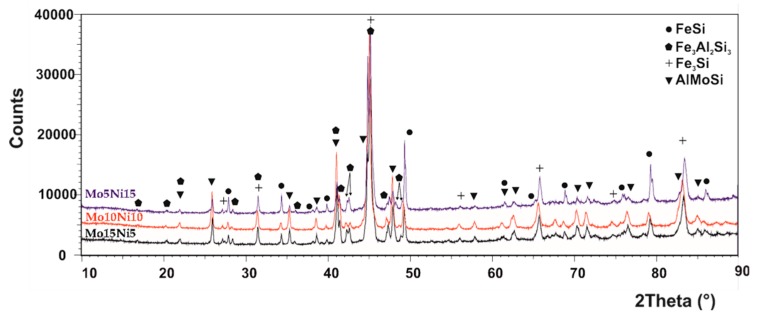
XRD diffraction patterns of the mechanical alloying and spark plasma sintering (MA + SPS) FeAl20Si20-Mo-Ni (wt %) alloys containing Mo and Ni in a range from 5–15 wt %.

The phase composition of all the MA + SPS alloys has been determined by the Rietveld X-ray diffraction analysis, and the patterns are shown in Figure 1. Accordingly to the results, all the prepared alloys were composed of two binary FeSi and Fe_3_Si phases and of two ternary Fe_3_Al_2_Si_3_ and AlMoSi phases, and the lattice parameters are shown in Table 1. Compared to the work of others [34,35,36], the short-term MA formed only intermetallic phases instead of solid solutions, which are created during much longer process durations. It was discovered that the different amount of the Mo and Ni addition did not change the phase compositions within the tested range of chemical compositions. All the phases were showing the presence of crystallites with average dimensions around 50 nm (Table 2). Only the FeSi phase in the MA + SPS FeAl20Si20Mo5Ni15 alloy contained larger crystallites with average dimensions of 100 nm. The volume fraction of the phases varied with the increasing content of Mo, favoring the formation of a ternary AlMoSi phase. Thus, the content of the AlMoSi phase increased from 8.5 wt % up to 23.0 wt %, mostly at the expanse of the FeSi phase. All of the mentioned phases were saturated with other elements, which slightly changed the lattice parameters when compared to the known values. 

In comparison to our previous work [37], the addition of Mo and Ni resulted in the formation of an AlMoSi phase, which depleted the content of the elements within the remaining phases. As a result, the lattice parameters of the FeSi, Fe_3_Si, and Al_2_Fe_3_Si_3_ phases were in the majority of the cases lower compared to the lattice parameters obtained for identical phases in the FeAl20Si20 alloy. These results also differed from results observed by others, which, e.g., either calculated the values of the Fe_3_Si phase as *a* = 0.5650 nm [38] or determined the values of Fe_3_Al_2_Si_3_ by an XRD measurement [39]. The observed change in the lattice parameters was caused by the partial substitution of elements that caused stress–strains in the lattice, changing its parameters. Such observations have been already mentioned in our previous work [37] and the works of others [16,40].

It should be noted that the increasing content of the AlMoSi phase was followed by a decrease of the FeSi phase, reducing from 32.5 wt % to 17.0 wt %, which corresponded to FeAl20Si20Mo15Ni5 alloy. Additionally, the peak width was almost the same in all the alloys, which coincided with microstructural observations that confirmed almost identical dimensions of phases, regardless of the chemical composition.

**Figure 2:**
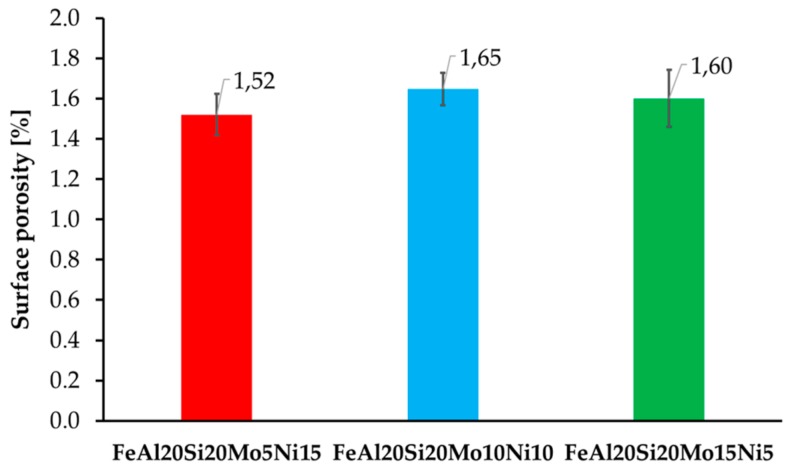
Surface porosity of the prepared MA + SPS FeAl20Si20-Mo-Ni alloys depending on the actual content of Mo and Ni within the alloy.

The surface porosity of the prepared MA + SPS alloys (Figure 2) has been determined by a threshold method for which the light micrographs prior etching were used. All of the prepared alloys were showing almost comparable porosity around 1.6%, which is almost three times higher than of the FeAl20Si20 alloys prepared by the same conditions [37]. The reason for the higher porosity might be found in the increased lattice stress–strains in present FeSi, Fe_3_Si, and Al_2_Fe_3_Si_3_ phases due to their enrichment by alloying elements. Besides, the formation of fine-grained AlMoSi phases also contributed to the overall strengthening of the material and further decreasing the plasticity during SPS compaction. Thus, the porosity could be only decreased using higher compaction temperatures, which would, on the other hand, promote a higher rate of microstructural coarsening that would deteriorate the overall mechanical properties. 

**Figure 3:**
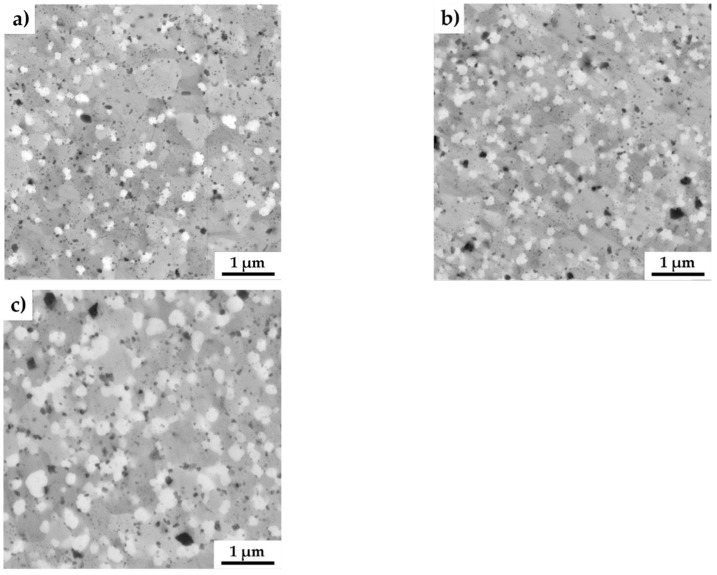
SEM micrographs of the MA + SPS FeAl20Si20-Mo-Ni alloys showing (**a**) FeAl20Si20Mo5Ni15; (**b**) FeAl20Si20Mo10Ni10; and (**c**) FeAl20Si20Mo15Ni5 alloys (a combination of BSE+SE detectors was used).

The SEM micrographs of all the MA + SPS alloys are shown in Figure 3. As can be seen, the MA + SPS alloys showed uniform microstructure with homogeneously distributed particles of intermetallic phases. These phases were mostly polyhedral in shape with various dimensions, as is shown. The present phases were roughly distinguished based on the physical background of the used backscattered electron detector, which displays elements with higher atomic number as bright areas, while lighter atoms manifest themselves as darker objects. Thus, based on the observations, three evident areas with different chemical content were discovered. The brightest rounded particles with an average diameter below 200 nm were containing Mo and thus corresponded to the AlMoSi phase. The bright gray and middle gray phases were showing sufficient brightness, suggesting the presence of elements such as Fe and Si, identifying themselves as FeSi or Fe_3_Si phases. The dark gray phases were showing the presence of light elements such as Al, and thus were initially identified as Fe_3_Al_2_Si_3_ phases. Among these phases, small dark and rounded objects were also observed. The particles could be either pores, which formed during etching in a reagent containing fluoride ions, or oxide particles. The origin of oxides particles might be found in the pre-oxidized powders, since the process of mechanical alloying was done in a protective Ar atmosphere. Comparing all the alloys used for MA, the lowest standard Gibbs energy of ΔG _298,16_ = –1584.0 kJ·mol^−1^ corresponded to the formation of Al_2_O_3_, followed by the value of Fe_3_O_4_ (ΔG _298,16_ = −1015.3 kJ·mol^−1^) [41]. When compared to other alloys, these oxides exhibit the highest affinity to oxygen and thus are the primary sources of contamination via oxygen. Among the oxidic particles, the presence of small dimples caused by fluorine ions, which were present in the etching solution, was observed. 

**Figure 4:**
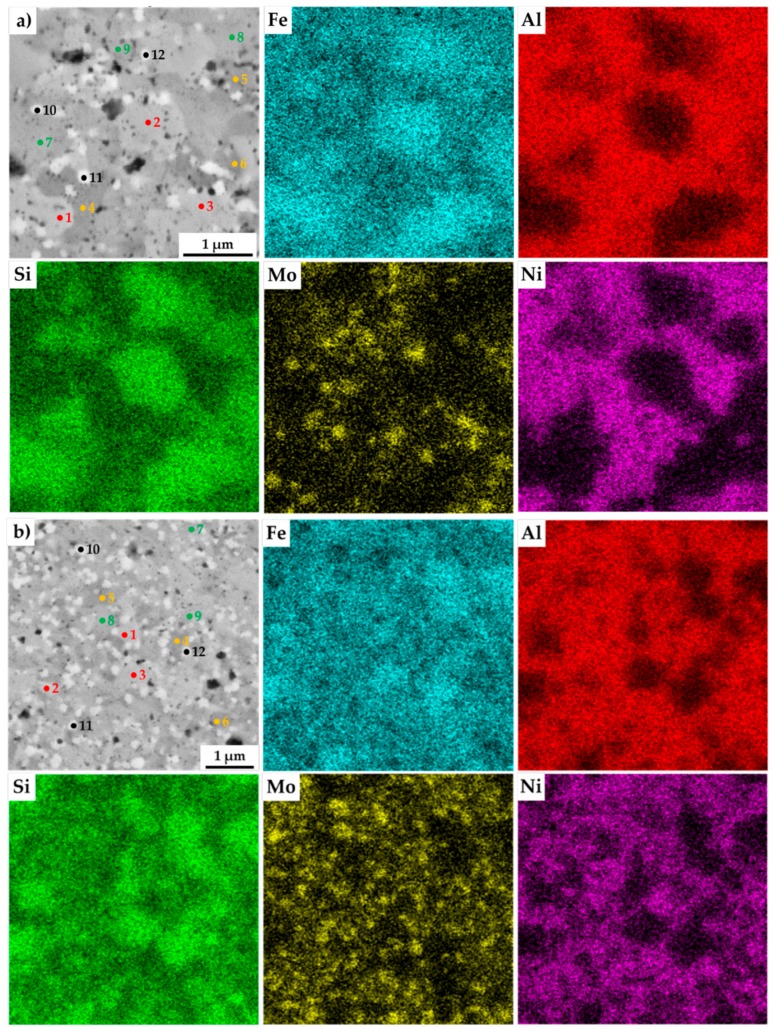

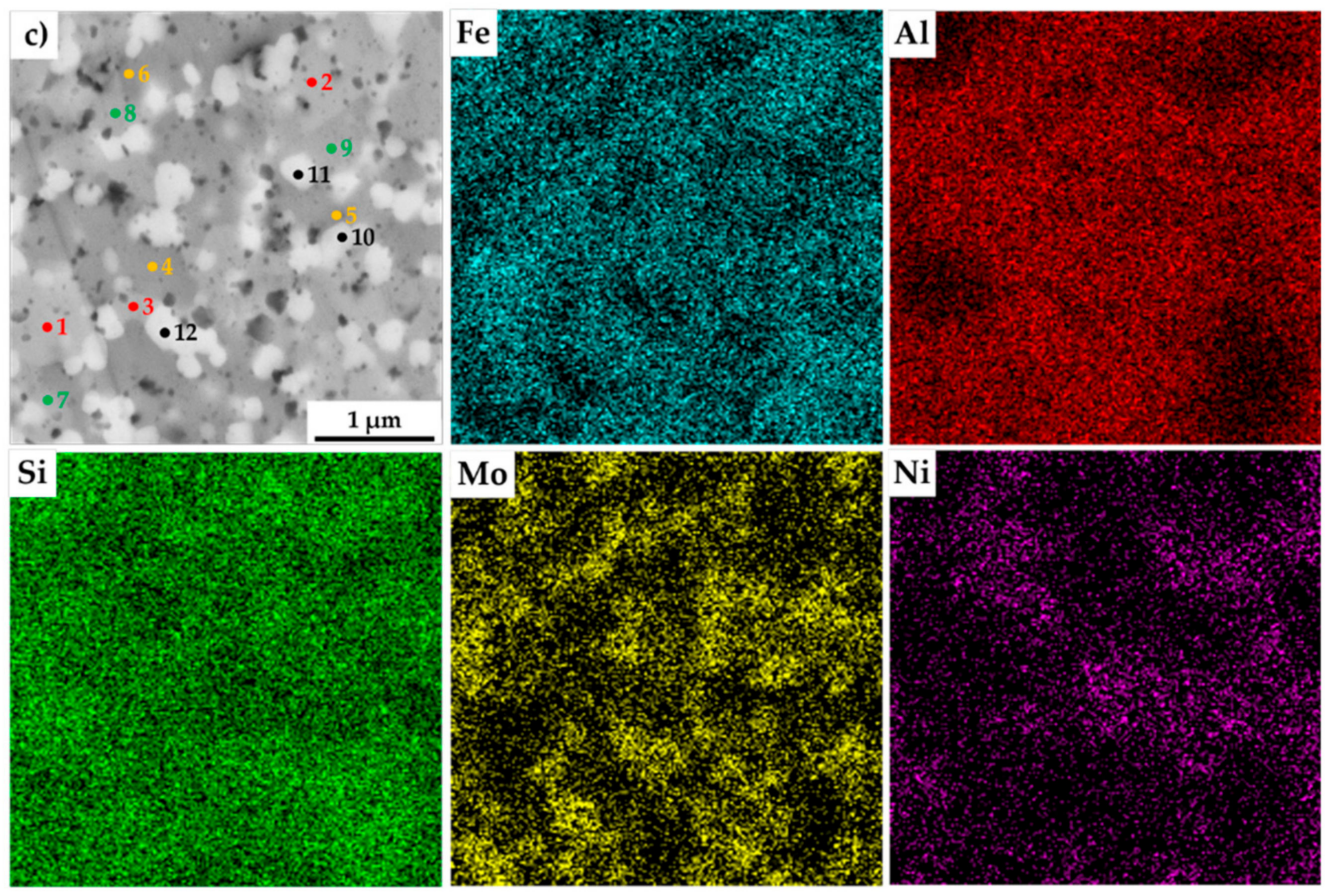
SEM+EDS element distribution maps of the MA+SPS: (**a**) FeAl20Si20Mo5Ni15; (**b**) FeAl20Si20Mo10Ni10; (**c**) FeAl20Si20Mo15Ni5 alloys with the marked places of chemical analysis, whose results are shown in Table 3.

The present phases were distinguished by SEM+EDS element distribution maps, as shown in Figure 4. The maps show large areas, which were enriched mainly in Fe and Si. On the other hand, the areas enriched in Al were also containing Ni. Nevertheless, the present phases were hardly distinguishable by the appearance of the element distribution map, since the elements often supersaturate the phases, exceeding the expected concentrations. Such observations are nothing unusual, considering that the preparation via MA can be briefly described as a non-equilibria process allowing the creation of phases that are enriched of other elements. Thus, these areas were analyzed by the SEM+EDS point analysis to determine the average chemical composition of present phases, whose results are shown in Table 3.

The results of the point analysis show the already mentioned enrichment of binary FeSi and Fe_3_Si phases by other alloying elements, namely of Al. Its concentration ranged in the FeSi phase, regardless of the chemical composition of the MA + SPS alloy, from 14.2 up to 16.4 at %. The same phase contained a significantly lower amount of Ni whose concentration decreased from 3.3 at % to 1.3 at %, reflecting the decreasing content of Ni in the MA + SPS alloy. On the other hand, the Fe_3_Si phase showed the supersaturation of Ni, whose lowest concentration of 8.5 at % further increased up to 19.3 at.% in the FeAl20Si20Mo5Ni15 alloy. Both the binary silicides contained only small amounts of Mo below 3.0 at %. The ternary Fe_3_Al_2_Si_3_ phase was showing a competing substitution of Ni and Mo, which had content that was changing concerning the chemical composition of the investigated alloy. Despite this, the AlMoSi phase showed, as the only one, almost constant chemical composition across all the investigated alloys. All of the present phases were showing the enrichment of other elements, while the chemical composition was shifted out of the equilibria state.

### 3.2. Mechanical Properties

**Figure 5:**
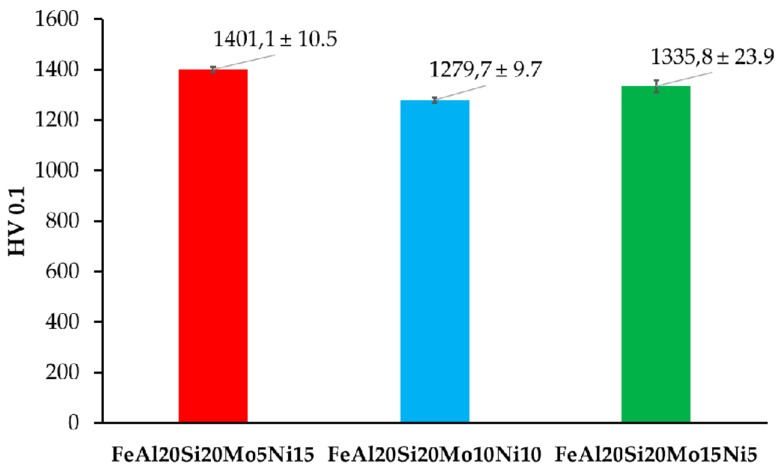
Vickers hardness of the prepared MA + SPS FeAl20Si20-Mo-Ni alloys.

The prepared alloys were after compaction via SPS tested for Vickers hardness, whose results are shown in Figure 5. As is shown, all the prepared MA + SPS alloys exhibited high hardnesses, which exceed those observed in only the ternary FeAl20Si20 alloy prepared in our previous research [37]. In direct comparison, the FeAl20Si20Mo5Ni15 exceeded the hardness of FeAl20Si20 alloy by almost more than 300 HV 0.1. Such an increase in hardness can be attributed to a higher content of especially FeSi and Fe_3_Al_2_Si_3_ phases which reached in total up to 58.5 wt % for the first alloy (see Table 2). These phases, respecting the order of their appearance, are known to exhibit hardnesses up to 958 HV and 1553 HV, respectively. The presence of the Fe3Si phase more than surely softened the alloy since exhibiting a maximal hardness of 514 HV [16,19]. 

However, this presumption corresponds to the results of the FeAl20Si20Mo15Ni5 alloy that showed the second-highest hardness, containing only 54 wt % of the previously mentioned phases. Thus, considering the phase fractions of each present phases (see Table 4), the highest contribution toward the hardness is caused by the ternary Fe_3_Al_2_Si_3_ phase. This presumption seems to be correct, since the content of this phase was lowest in the case of FeAl20Si20Mo10Ni alloy, which also showed the lowest hardness 1279.7 ± 9.7 HV 0.1 of all the alloys. Besides, the deformation strengthening of the present phases needs to be also taken into account, among which at least the FeSi has been reported to achieve plastic deformation under extreme conditions [42,43]. Besides, the presence of the oxidic particles within the grains of present phases might contribute to the overall strengthening of the prepared MA + SPS alloys. Thus, the ultrahigh hardness of 1401 HV 0.1 of the FeAl20Si20Mo5Ni15 alloy has been achieved by a synergic contribution of all the above-mentioned effects, exceeding the hardness of laser-cladding Fe–Al–Si layers (560 HV 0.1) [44] almost three times over and almost two times over compared to those prepared by SHS reaction (860 HV 5) [15].

**Figure 6:**
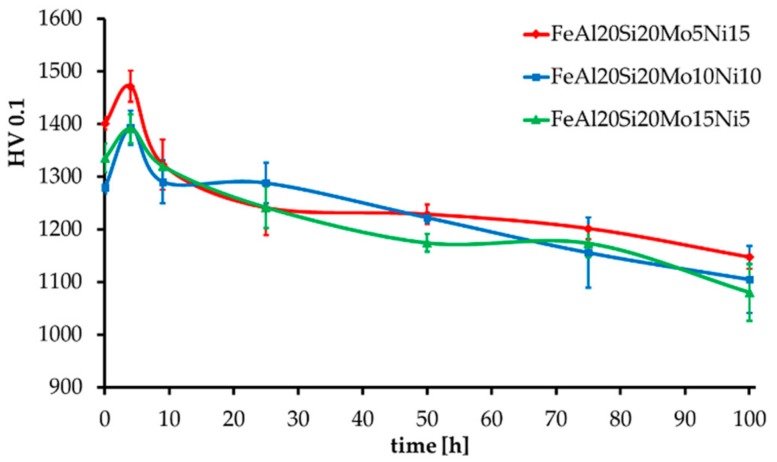
Thermal stability of the MA + SPS alloys expressed as HV 0.1 change during long-term annealing at 800 °C.

The MA + SPS alloys have also been tested for thermal stability, which was expressed as hardness change during long-term annealing at 800 °C, as shown in Figure 6. All the alloys showed an initial increase in hardness by approximately 60 HV 0.1, followed by a slow decrease in hardness as the duration of annealing prolonged up to a total of 100 h. In the end, all the alloys showed, considering the confidence intervals, almost identical values of hardness reaching over 1100 HV 0.1. The highest hardness prior and after the tests was obtained by the FeAl20Si20Mo5Ni15 alloy, which contained the highest volume fraction of FeSi, Fe_3_Si, and Fe_3_Al_2_Si_3_ phases reaching up to 91.5 wt %. During the first 4 h of annealing, the hardness increased probably due to a formation of precipitates within the material, which further either dissolved or coarsened, reducing its strengthening contribution toward the alloy. Such a presumption might be supported by the already-mentioned enrichments of present phases by other elements due to a non-equilibria preparation process. A further decrease in hardness was caused by the microstructural coarsening of each constituent. 

**Figure 7:**
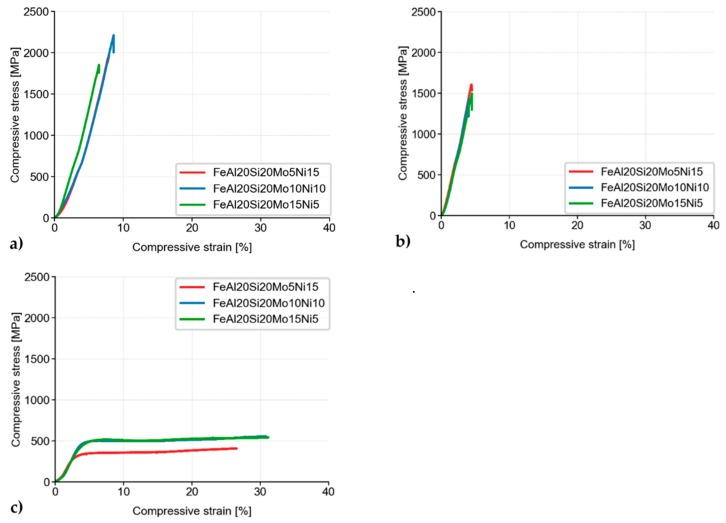
Compressive stress–strain curves of MA + SPS alloys at (**a**) laboratory temperature; (**b**) laboratory temperature after 100 h of annealing at 800 °C; and (**c**) a temperature of 800 °C.

Besides, the MA + SPS alloys have been compressively tested either at laboratory temperature (Figure 7a), at a laboratory temperature after 100 h of annealing at 800 °C (Figure 7b), or at an elevated temperature of 800 °C (Figure 7c). As is shown, all the alloys showed ultrahigh ultimate compressive strengths (UCS) at laboratory temperature, among which the FeAl20Si20Mo10Ni10 alloy reached the highest UCS of approximately 2200 MPa, outperforming the second-best FeAl20Si20Mo5Ni15 alloy by more than 200 MPa. 

When tested after 100 h annealing at 800 °C, all of the MA + SPS alloys softened, reducing its UCS down to approximately 1600 MPa. The observed decrease in the UCS value was in good agreement with the already observed hardness decrease due to a coarsening of present phases. The observed differences between each alloy were almost negligible, although the highest UCS of 1600 MPa was achieved in the case of the FeAl20Si20Mo5Ni15 alloy, which also showed the highest hardness after annealing. 

The MA + SPS alloys were also tested at an elevated temperature of 800 °C (Figure 7c). During these tests, all of the previously brittle alloys changed their behavior, exhibiting significant plasticity due to the activation of non-discrete dislocation movements. Although the tests were done at elevated temperature, the FeAl20Si20Mo10Ni10 and FeAl20Si20Mo15Ni5 showed almost identical values of compressive yield strength (CYS), which were 428 and 437 MPa, respectively. 

**Figure 8:**
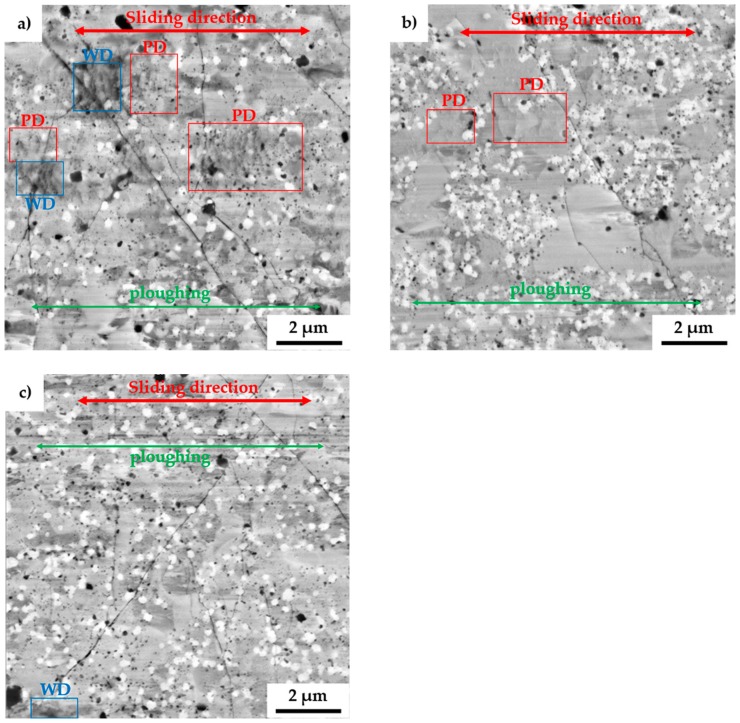
SEM micrographs of the wear tracks of the MA + SPS: (**a**) FeAl20Si20Mo5Ni15; (**b**) FeAl20Si20Mo10Ni10; and (**c**) FeAl20Si20Mo15Ni5 alloys. (PD – plastic deformation; WD – wear debris).

To fully describe the mechanical properties, the alloys were also tested by the pin-on-disc method to determine wear-related characteristics. After the tests, the morphology of wear tracks was observed with SEM, as is shown in Figure 8. The wear tracks show the presence of thermally induced microcracks whose origins were randomly distributed across all the present phases. Among that, some areas were showing the presence of wrinkles, which were pointed at localized plastic deformation (PD) within the wear track. Such behavior comes along with the already observed plastic deformation during compressive tests at elevated temperature. However, since the wear tests are highly localized, the heat dissipation in the bulk of the material is enormous, allowing the creation of thermally induced cracking of the surface. Besides, the wear debris (WD) at the ends of the wear tracks were composed of oxides, which confirms the presumption. These particles, which were mostly composed of different oxides, were also randomly present in the wear track as is shown, especially in Figure 8a. 

All the MA + SPS alloys were showing exceptional wear resistance with a wear rate that was almost one magnitude lower than that of the reference tool steel 1.3343 (Table 5). Among that, the friction coefficients of these materials were significantly lower and much steadier than those of the reference tool steels. The lowest friction coefficient of 0.446 was achieved in the case of FeAl20Si20Mo10Ni10 alloy, which also exhibited the lowest wear rate of 3.14 10^−6^ mm^3^·N^−1^·m^−1^. On the other hand, the reference tool steels were showing significantly higher friction coefficients as well as higher wear rates reaching up to 1.46 10^−5^ mm^3^·N^−1^·m^−1^. Such a high friction coefficient indicated high tangential forces between the ball and tested materials, which need to be overcome to maintain the movement during the wear test. As a result, a lot of energy dissipates during the intensive plastic deformation of sublayers beneath the sliding ball. As a result, the wear track of the tool steels contained deep and wide grooves from ploughing the released particles, enhancing the three-body abrasion. 

Compared to that, the MA + SPS alloys showed a rather shallow profile of the wear track with only minor traces of ploughing, as is shown in Figure 8. The grooves were present in all the observed phases, implying that the cohesion between the phases was sufficient, and none of them chipped off, acting as a powerful abrasive medium that would significantly increase the wear rate. Such behavior of the MA + SPS alloys was responsible for achieving a low friction coefficient, as was discovered. 

These excellent results of wear resistance were a direct consequence of the phase composition of all the MA + SPS alloys containing binary and ternary silicides, which comes along the ultrafine-grained microstructure and good cohesion of powder particles as well as of the present phases, which did not tend to chip. Considering the high hardness of all the MA + SPS alloys, the primary wear mechanism seems to be oxidation wear together with a minor contribution of abrasive wear. This presumption is supported by the presence of wrinkles pointing at the plastic deformation that are these materials capable of only at elevated temperatures as well as by the presence of oxides found within the wear track or at the end of the wear track. 

### 3.3. Oxidation Resistance

**Figure 9:**
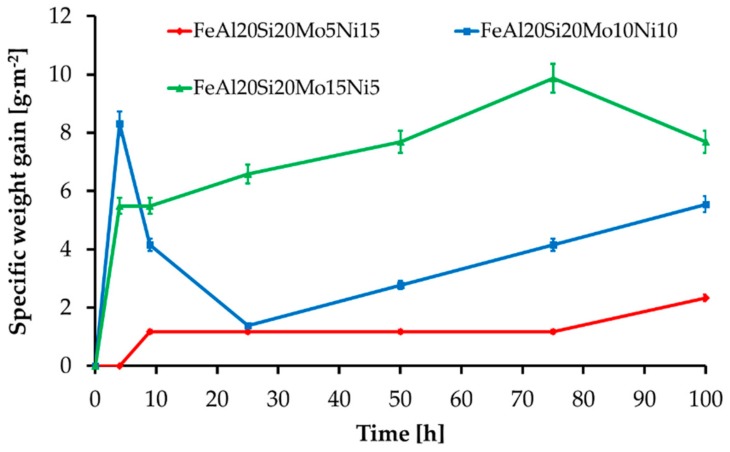
Oxidation kinetics of the MA + SPS FeAl20Si20-Mo-Ni alloys during annealing at 800 °C.

The MA + SPS alloys have also been investigated for cyclic oxidation resistance at 800 °C during the early beginnings in standard atmosphere. All the alloys formed a layer made of oxidic products without any traces of delamination. The layer growth during the first hours of annealing manifested as a steep weight increase followed by a decrease in weight gain speed, since the layer has been effectively shielding the material, slowing the kinetics of oxidation. The initial steps of the oxide layer formation were reaction controlled, while a steep decrease in a weight gain suggested the formation and growth of a protective oxidic membrane whose presence further slowed oxidation. Thus, as the time of oxidation prolonged, the kinetics became controlled by oxygen diffusion through the developed oxidic layer. As is shown in Figure 9, all of the MA + SPS alloys showed exceptional oxidation resistance as the time of the test prolonged, which corresponded to a formation of the protective oxidic barrier. The highest oxidation resistance was observed in the case of FeAl20Si20Mo5Ni15 alloy, which during the first 4 h of cyclic oxidation did not create any traces of an oxidic layer. As the duration of the oxidation test prolonged, the same alloy developed a compact layer of oxides which effectively shielded the material, resulting in the lowest specific weight gain of approximately 2.5 g·m^−2^ among all tested alloys. However, the other MA + SPS alloys showed somewhat higher weight gains reaching up to 10 g·m^−2^, which are still good enough. It should be noted that due to the limited dimensions of the samples, the weight gains were typical in the order of mg. 

An oblique cross-section has been prepared to display the oxidic layer sufficiently. The thickness of the present oxidic layer (Table 6) obtained via the oblique cross-section has been calculated using Equation (1):

(1) $$d_{r}=d_{m}\cdot \sin\left(\tan^{-1}\left(\frac{r_{s}}{l}\right)\right)$$

where *d_r_* is the real thickness of the oxidic layer, *d_m_* is the measured thickness on the oblique cross-section; *r_s_* is the diameter of used support, and *l* is the distance between the support and the oxidic layer. As is shown in Table 6, the real thickness of the oxidic layers after 100 h of oxidation done at 800 °C was around 1 µm. 

The thicknesses of the present oxidic layers observed on oblique cross-sections were after 100 h of cyclic oxidation almost identical, reaching approximately 1 µm.

**Figure 10:**
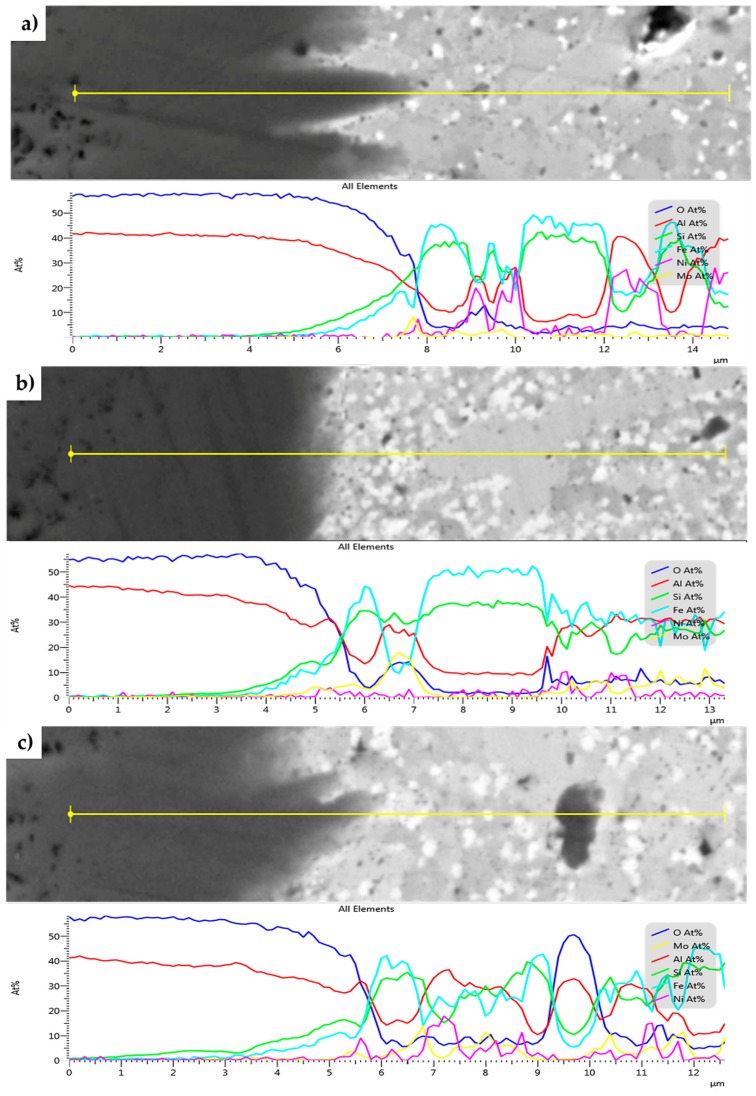
SEM+EDS linescans across the oxide layers formed on the MA + SPS: (**a**) FeAl20Si20Mo5Ni15; (**b**) FeAl20Si20Mo10Ni10; and (**c**) FeAl20Si20Mo15Ni5 alloys after 100 h of cyclic oxidation at 800 °C.

The SEM + EDS line profiles across the present oxidic layer for all the MA + SPS alloys are shown in Figure 10. From the EDS line profiles, it is visible that the oxidic layer is on the outside containing only Al and O, whose atomic ratios corresponds to Al_2_O_3_. Its presence is visible throughout the entire oxidic layer. However, the content of Al_2_O_3_ changes near the oxide–alloy interface, while the content of Si and Fe increases. The ratio of present oxides is changing, favoring the presence of SiO_2_ and FeO-based oxides. The thickness of this sublayer, where the content of SiO_2_ and FeO-based oxides increased, was approximately 0.5 µm for the first two alloys. 

On the other hand, the FeAl20Si20Mo15Ni15 alloy showed an increased concentration of Si already in a distance of 1 µm from the interface of the environment oxidic layer. The increasing concentration of Si was later followed by an increasing concentration of Fe as the distance increased. The presence of Si in the deeper parts of the oxidic layer significantly improved the oxidation resistance, outperforming those of the other tested MA + SPS alloys.

The formation of Al_2_O_3_ layers depleted the sub-areas beneath the layer, showing the presence of phases whose concentration of elements corresponded to the FeSi phase, still saturated, among other elements, with Al reaching up to 15 at.%. Such a finding was already discussed in the work of [45], who proposed and also verified that the increased diffusion of Al through the newly developed barrier depleted the present intermetallic phases from Al, resulting in a formation of silicides. Additionally, some of the previously mentioned black areas were showing an increased concentration of Al and O, whose ratios corresponded to Al_2_O_3_. The origin of these particles can be found in partially pre-oxidized powder particles of Al and others, which during the MA formed the most thermodynamically stable Al_2_O_3_ product. 

## 4. Conclusions

A combination of mechanical alloying and compaction via spark plasma sintering successfully prepared the FeAl20Si20-Mo-Ni alloys containing from 5–15 wt % of the alloying elements. Prepared alloys showed a uniform microstructure composed of four phases, namely of FeSi, Fe_3_Si, Fe_3_Al_2_Si_3_, and AlMoSi phases. Formation of the AlMoSi phase increased the hardness and compressive strength of the alloys. It was found that increasing the amount of Mo in the alloy reduced the fraction of FeSi phase at the expanse of the newly created AlMoSi phase. All of the present phases were enriched with alloying elements, increasing the lattice stress–strains due to their deformation. The main contribution toward the hardness of the alloys was, among the MA process itself, caused by Fe_3_Al_2_Si_3_, whose content together with the second-hardest FeSi phase reached in the FeAl20Si20Mo5Ni15 up to 58.5 wt %. 

As a direct correlation with the hardness, the investigated alloys outperformed the reference tool steels by more than one order of magnitude regarding the wear rate while exhibiting lower friction coefficients. The primary wear mechanism was found to be oxidation wear, which subsequently allowed abrasive wear due to the presence of oxidic debris. During the cyclic oxidation tests, all the alloys showed exceptional oxidation resistance while creating a compact layer of oxidic products that was mainly composed of Al_2_O_3_ without any traces of delamination. Furthermore, it was found that the highest oxidation resistance of the FeAl20Si20Mo5Ni15 was caused by slightly different ratios of oxides present in the layer. The oxidic layer was initially composed of Al_2_O_3_ at the environment–layer interface and slightly changed, revealing an increase in the content of Si followed by Fe.

## Figures and Tables

**Table 1:** Lattice parameters determined by the XRD analysis of present phases identified in the MA + SPS FeAl20Si20-Mo-Ni alloys.

Phases	Lattice Parameters					
	*a* (nm)	*b* (nm)	*c* (nm)	α (°)	β (°)	γ (°)
FeSi	0.4519 ± 0.0001	–	–	–	–	–
Fe_3_Al_2_Si_3_	0.4668 ± 0.0003	0.6325 ± 0.0004	0.7486 ± 0.0004	101.12 ± 0.04	105.88 ± 0.04	101.32 ± 0.04
Fe_3_Si	0.5637 ± 0.0001	–	–	–	–	–
AlMoSi	0.4655 ± 0.0002	–	0.6532 ± 0.0004	–	–	–
FeSi	0.4530 ± 0.0001	–	–	–	–	–
Fe_3_Al_2_Si_3_	0.4707 ± 0.0003	0.6222 ± 0.0004	0.7472 ± 0.0004	100.60 ± 0.04	105.36 ± 0.05	101.87 ± 0.05
Fe_3_Si	0.5688 ± 0.0001	–	–	–	–	–
AlMoSi	0.4668 ± 0.0001	–	0.6538 ± 0.0001	–	–	–
FeSi	0.4530 ± 0.0001	–	–	–	–	–
Fe_3_Al_2_Si_3_	0.4686 ± 0.0002	0.6320 ± 0.0002	0.7504 ± 0.0003	100.95 ± 0.01	105.70 ± 0.02	101.54 ± 0.03
Fe_3_Si	0.5684 ± 0.0001	–	–	–	–	–
AlMoSi	0.4665 ± 0.0001	–	0.6539 ± 0.0002	–	–	–

**Table 2:** The phase parameters, phase fractions, and crystallite sizes determined by Rietveld analysis.

Alloy	Phases	Space Group	Wt %	Crystallite Size (nm)
FeAl20Si20Mo5Ni15	FeSi	*P*2_1_3	32.5 ± 0.1	100
	Fe_3_Al_2_Si_3_	*P* $\bar{1}$	24.5 ± 0.1	≈ 50
	Fe_3_Si	*Fm* $\bar{3}$ *m*	34.5 ± 0.1	43
	AlMoSi	*P*6_2_22	8.5 ± 0.1	63
FeAl20Si20Mo10Ni10	FeSi	*P*2_1_3	30.0 ± 0.1	65
	Fe_3_Al_2_Si_3_	*P* $\bar{1}$	18.5 ± 0.2	≈ 50
	Fe_3_Si	*Fm* $\bar{3}$ *m*	39.0 ± 0.1	35
	AlMoSi	*P*6_2_22	12.5 ± 0.1	≈ 60
FeAl20Si20Mo15Ni5	FeSi	*P*2_1_3	17.0 ± 0.1	50
	Fe_3_Al_2_Si_3_	*P* $\bar{1}$	27.0 ± 0.2	≈ 50
	Fe_3_Si	*Fm* $\bar{3}$ *m*	33.0 ± 0.1	40
	AlMoSi	*P*6_2_22	23.0 ± 0.1	55

**Table 3:** Results of the SEM+EDS analysis of the points marked in Figure 4.

Alloy	Points	Average Chemical Compositon (at %)					Corresponding Phase
		Al	Si	Fe	Ni	Mo	
**FeAl20Si20Mo5Ni15**	**1–3**	14.4 ± 3.9	38.1 ± 3.9	43.6 ± 1.0	3.3 ± 1.4	0.6 ± 0.3	**FeSi**
	**4–6**	31.7 ± 2.1	28.3 ± 2.4	34.1 ± 1.2	5.1 ± 3.9	0.9 ± 0.7	**Fe_3_Al_2_Si_3_**
	**7–9**	34.9 ± 0.8	20.9 ± 0,2	23.2 ± 0.2	19.3 ± 0.1	1.3 ± 0.1	**Fe_3_Si**
	**10–12**	31.5 ± 1.6	36.2 ± 4.2	14.7 ± 0.5	4.5 ± 3.8	13.0 ± 1.5	**AlMoSi**
**FeAl20Si20Mo10Ni10**	**1–3**	14.2 ± 3.4	37.1 ± 2.1	45.0 ± 1.2	2.1 ± 0.7	1.5 ± 0.6	**FeSi**
	**4–6**	33.8 ± 1.9	26.0 ± 1.0	34.8 ± 0.1	3.1 ± 0.9	2.2 ± 0.2	**Fe_3_Al_2_Si_3_**
	**7–9**	34.0 ± 0.3	20.9 ± 0.3	30.9 ± 0.8	12.7 ± 0.3	1.6 ± 0.2	**Fe_3_Si**
	**10–12**	27.0 ± 0.3	38.5 ± 2.5	15.5 ± 5.4	3.8 ± 0.1	15.2 ± 3.3	**AlMoSi**
**FeAl20Si20Mo15Ni5**	**1–3**	16.4 ± 2.3	36.3 ± 2.3	43.0 ± 2.5	1.3 ± 0.7	3.0 ± 1.8	**FeSi**
	**4–6**	34.0 ± 0.8	30.8 ± 5.0	27.2 ± 10.1	0.8 ± 1.2	7.8 ± 7.1	**Fe_3_Al_2_Si_3_**
	**7–9**	35.5 ± 0.1	22.0 ± 1.2	33.2 ± 0.4	8.5 ± 0.7	1.9 ± 0.2	**Fe_3_Si**
	**10–12**	29.8 ± 0.8	37.4 ± 1.2	15.3 ± 1.2	1.3 ± 1.1	16.2 ± 1.7	**AlMoSi**

**Table 4:** Phase fractions and their correlation with the measured hardness of the MA + SPS alloys. *(differences from FeAl20Si20Mo5Ni15 are shown in brackets)*.

Phases	Phase Fractions in the FeAl20Si20-Mo-Ni alloy [%]		
	Mo5Ni15	Mo10Ni10	Mo15Ni5
FeSi	32.5	30.0 **(−2.5)**	17.0 **(−15.5)**
Fe_3_Si	34.5	39.0 **(+4.5)**	33.0 **(−1.5)**
Fe_3_Al_2_Si_3_	24.5	18.5 **(−6.0)**	27.0 **(+2.5)**
AlMoSi	8.5	12.5 **(+4.0)**	23.0 **(+14.5)**
HV 0.1	1401.1 ± 10.5	1279.7 ± 9.7	1335.8 ± 23.9

**Table 5:** Results of the wear tests done at laboratory temperatures of all the MA + SPS alloys and two references *(Ra – surface roughness)*.

Alloy	Ra (µm)	Wear (mm^3^·N^−1^·m^−1^)	RSD (±)	Friction Coefficient (–)
FeAl20Si20Mo5Ni15	0.0166	5.97 × 10^−6^	3.33 × 10^−7^	0.495
FeAl20Si20Mo10Ni10	0.0062	3.14 × 10^−6^	3.00 × 10^−7^	0.446
FeAl20Si20Mo15Ni5	0.0139	5.68 × 10^−6^	4.48 × 10^−7^	0.498
Steel 1.2379	0.0096	1.46 × 10^−5^	1.51 × 10^−6^	0.732
Steel 1.3343	0.0080	2.84 × 10^−6^	1.93 × 10^−7^	0.669

**Table 6:** Real thickness of an oxidic layer after 100 h at 800 °C observed on an oblique cross-section calculated using Equation (1).

Alloy	Thickness of Oxidic Layer (µm)
FeAl20Si20Mo5Ni15	0.99
FeAl20Si20Mo10Ni10	1.13
FeAl20Si20Mo15Ni5	0.83
